# Association of physical activity, sedentary behavior and stroke in older adults

**DOI:** 10.3389/fpubh.2024.1484765

**Published:** 2024-12-20

**Authors:** Long Bai, Zongliang Wen, Xuebing Yan, Shenqin Wu, Jialin Chen

**Affiliations:** ^1^School of Public Health, Xuzhou Medical University, Xuzhou, China; ^2^School of Management, Xuzhou Medical University, Xuzhou, China; ^3^Affiliated Hospital of Xuzhou Medical University, Xuzhou, China

**Keywords:** NHANES, sedentary behavior, physical activity, stroke, mediation analysis

## Abstract

**Objective:**

The aim of this study was to investigate the relationship between physical activity, sedentary behavior and stroke in people aged 60 years and older.

**Methods:**

The study included 3,010 participants aged 60 and older from the National Health and Nutrition Examination Survey (NHANES). Data on sedentary behavior, physical activity and stroke were obtained through questionnaires. Statistical analyses were performed using a complex multistage sampling design and weighted multivariate logistic regression. Smoothed curve fitting and threshold effects analyses were used to explore non-linear relationships between physical activity, sedentary behavior and stroke.

**Results:**

There were 244 (7.53%) participants aged 60 years and older who had experienced a stroke. After adjusting for all covariates, physical activity, sedentary behavior and stroke were significantly associated [OR (95% CI) for physical activity: 0.622 (0.443, 0.875), *p* = 0.009; OR (95% CI) for sedentary behavior: 2.602 (1.557, 4.348), *p* = 0.003]. C-reactive protein mediated the association between sedentary behavior and stroke among older adults, with a mediation of 3.64%.

**Conclusion:**

In people aged 60 years and older, sedentary behavior was positively associated with stroke, whereas physical activity was negatively associated with stroke, and C-reactive protein mediated the relationship between sedentary behavior and stroke.

## Introduction

Stroke is a clinical syndrome caused by focal or systemic brain damage that lasts more than 24 h or results in death. In highly developed countries, stroke is the third most common cause of adult death, the second most common cause of dementia, and the most common cause of disability (1). Lifestyle, hypertension, hyperlipidemia, blood glucose, obesity, and obstructive sleep apnea are risk factors for stroke (2). Stroke is the leading cause of disability worldwide and the second leading cause of death. The Global Stroke Factsheet released in 2022 reveals that lifetime risk of developing a stroke has increased by 50% over the last 17 years and now 1 in 4 people is estimated to have a stroke in their lifetime. In 2021, stroke was the third most common GBD level 3 cause of death (7.3 million [95% UI 6.6–7.8] deaths; 10.7% [9.8–11.3] of all deaths) after ischemic heart disease and COVID-19, and the fourth most common cause of DALYs (160.5 million [147.8–171.6] DALYs; 5.6% [5.0–6.1] of all DALYs) (3).

There is a large body of evidence that lifestyle factors significantly affect the incidence of stroke and that reducing sedentary time and increasing exercise are necessary changes that can benefit people (4, 5). Studies have pointed to a significantly greater prevalence of physical inactivity among stroke survivors (6). Inflammation is a response triggered by damage to living tissue. Growing evidence for the double-edged role of the immune system in the pathophysiology of stroke (7). A study points to inflammatory cytokines and cells as potential markers for stroke patients in intensive care units (8). It has also been suggested that elevated baseline C-reactive protein levels are associated with an increased risk of ischemic stroke (9). A meta-analysis of individual participant data suggests that blood markers of inflammation are independently associated with vascular recurrence after a stroke (10).

Physical activity is a potential modulator of the inflammatory process, which reduces inflammation and thus reduces the incidence of many diseases, such as multiple sclerosis (11). Other evidence also suggests that increased exercise is associated with reduced inflammation (12, 13). Sedentary behavior is associated with increased inflammation in the body. Sedentary behavior causes significant pro-inflammatory effects (14). Sedentary men with metabolic syndrome have reduced inflammation after an exercise intervention, as evidenced by a reduction in hs-CRP levels (15).

This study examined the relationship between physical activity, sedentary behavior and stroke and assessed whether C-reactive protein could act as a mediator to mediate the relationship between physical activity, sedentary behavior and stroke by surveying a study population aged 60 years and older from the National Health and Nutrition Examination Survey (NHANES) over the past 4 years (2007–2010).

## Materials and methods

### Study population

**Figure 1:**
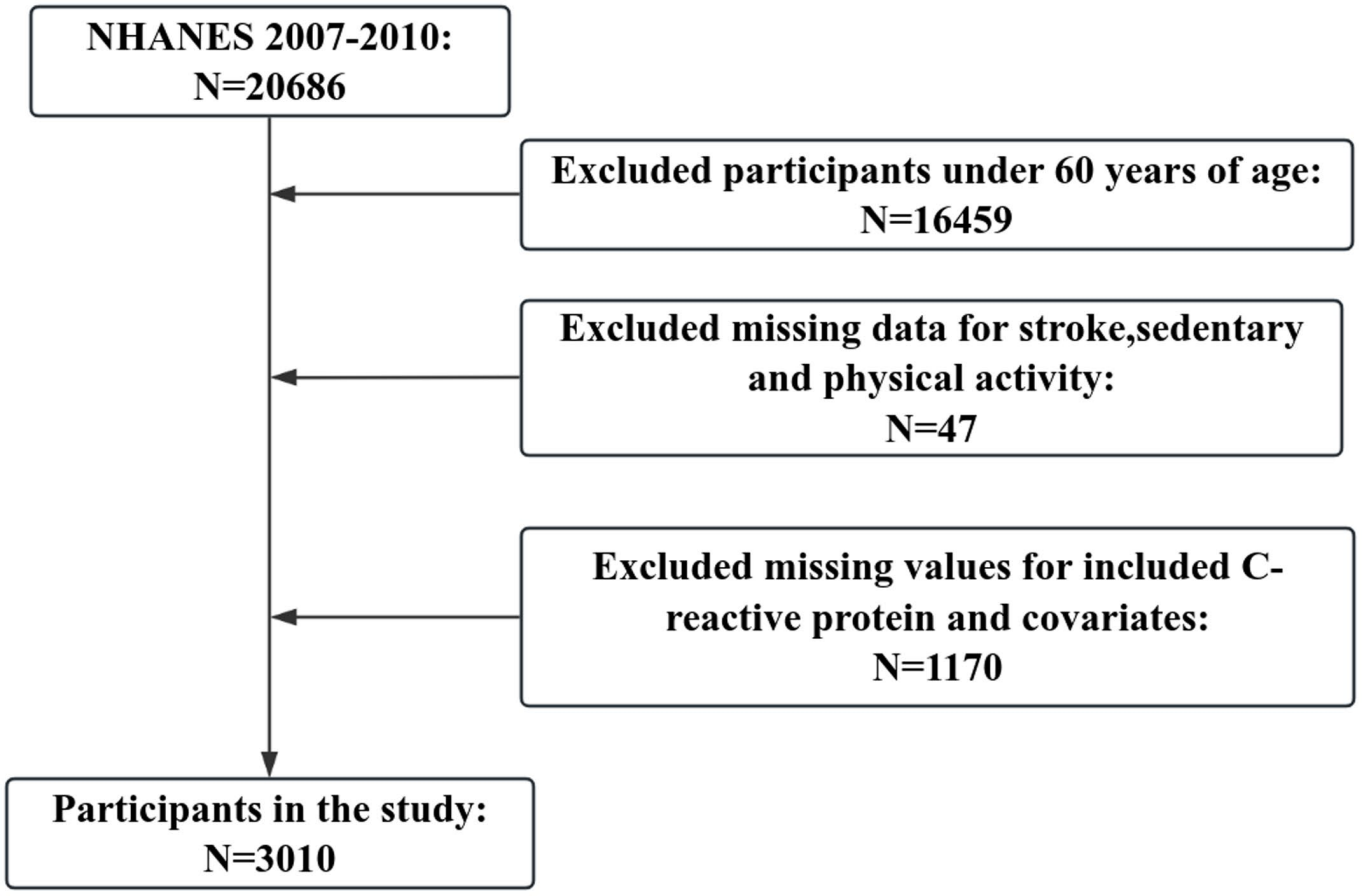
Flowchart of study design and exclusion of participants from the study.

The data analyzed in this study comes from the National Health and Nutrition Examination Survey (NHANES), a comprehensive population-based survey designed to collect data on the American civilian population. Since 1999, NHANES has collected data on approximately 10,000 individuals in 2-year cycles and has used a multistage probability sampling design to produce a representative sample of Americans in non-institutionalized households. We used data from two cycles in NHANES (2007–2008, 2009–2010). Participants with missing stroke information and invalid responses were excluded from data merging, while samples with missing basic information and C-reactive protein were excluded, resulting in 3,010 participants being included in our study (Figure 1).

### Assessment of stroke outcome

The purpose of this study, we defined stroke outcome as a self-reported positive diagnosis of stroke by a physician. All participants completed a disease-related health status questionnaire before their physical examination. Study participants were asked, “Has a doctor or other health professional ever told you that you had a stroke?” A “yes” answer to this question was coded as stroke-positive, and a “no” answer was coded as stroke-negative (16).

### Measurement of C-reactive protein

This method quantifies CRP by a latacy-enhanced turbidimetric method. For CRP quantification, a particle consisting of a polystyrene core and a hydrophilic shell was covalently linked to an anti-CRP antibody. Data reduction of the signal was performed by data reduction of the signal on the calibration curve using a storable logit-log function. Quantitative CRP was measured on a Behring nephelometer. The specific process can be found at https://wwwn.cdc.gov/Nchs/Nhanes/2009-2010/CRP_F.htm.

### Assessment of sedentary behavior and physical activity

The Physical Activity Questionnaire (prefix PAQ) is based on the Global Physical Activity Questionnaire (GPAQ) and includes problematic activities related to daily living, leisure time activities and sedentary activities. PA information was collected using a global PA questionnaire based on the one created by the World Health Organization (WHO) (17). Participants were asked to report their PA behaviors in the past 30 days^1^. Levels of three PA types were examined: strenuous work activity/recreational activity, moderate work activity/recreational activity, and walking/cycling activity. The number of days per week they engaged in each type of PA in a typical week was reported and the amount of time (in minutes) spent on that type of activity during the day. The frequency and duration of these activities were used to calculate weekly metabolic equivalent (MET) estimates. NHANES provides MET corresponding to each activity category to determine activity intensity.

First, Met/week was calculated by multiplying the total number of minutes per week for each activity by the NHANES-recommended MET value, PA (MET-minutes/week) = MET × weekly frequency × duration of each physical activity (18), and the sum of all activities was calculated by summing over all activity categories. Next, respondents were categorized according to the US PA guidelines for adherence (moderate-intensity PA for adults should be performed for 150 min per week [equivalent to 600 min/week]). Participants were categorized as physically inactive individuals (600 < MET minutes/week, less than PA recommendations) and physically active individuals (≥600 MET minutes/week, PA recommendations) (19). The NHANES Sedentary Behavior Assessment is a self-report question. Participants heard questions about how much time in a typical day you usually spend sitting or lying down at work, at home, or at school. This included sitting at a desk, with friends, traveling by car, bus or train, reading, playing cards, watching TV, or using a computer. Self-reported sitting or lying down time is divided into 4 levels (<4 h/d, 4–<6 h/d, 6–8 h/d, and >8 h/d) (20).

### Covariates

The following variables were included according to the purpose of the study. Gender (male/female), age (20–39, 40–59, and 60–80), race (Mexican American, other Hispanic, non-Hispanic white, non-Hispanic black, and other race), educational (<high school, high school, and >high school) and marital status (not living alone [Married/Living with Partner]/living alone [Widowed/Divorced/Separated/Never married]). Other confounding variables included smoking (defined as having smoked at least 100 cigarettes in a lifetime) and alcohol consumption (defined as having had at least 12 drinks in a year). Diabetes and hypertension were derived from self-reported physician diagnoses (yes/no). The body mass index is categorized into three categories (<25.0, 25.0-30.0, ≥30.0), and the poverty income ratio is an index of the ratio of household income to poverty.

### Statistical analyses

Given the complex multi-stage (stratified and whole group) sampling design of NHANES, appropriate weights were used. Empowerstats^2^ was used for weighted analyses. Sample weights for the Mobile Examination Centre (MEC) interviews were reweighted to combine the total NHANES survey data for the 4 years from 2007 to 2010. For baseline characterization, weighted means (95% CI) were used for continuous variables, observations and percentages (weighted) were used for categorical variables, *p*-values were calculated using appropriate weights, weighted linear regression was used for continuous variables, and weighted chi-square tests were used for categorical variables. Multivariate logistic regression analyses of the association between physical activity, sedentary behavior and stroke were performed and 95% confidence interval (CI) and odds ratio (OR) were calculated. Variables in the crude models were unadjusted; model 1 was adjusted for gender, age, and race, and model 2 was adjusted for gender, age, race, education, marital status, smoking status, drinking status, hypertension, diabetes, body mass index, and poverty income ratio. Mediation effects analyses were calculated using EmpowerStats (see Footnote 2) software. Mediated effects analyses allow us to calculate mediating effects and are an ideal strategy for elucidating pathways and providing statistical evidence for mechanistic analyses. In this study, direct effects represent associations between sedentary behavior, physical activity and stroke; indirect effects, i.e., associations between sedentary behavior, physical activity and stroke are mediated by inflammatory markers; and mediation ratios represent the percentage of mediated effects. *p* < 0.05 indicates a significant difference.

## Results

### Baseline characteristics of the study population

Table 1 shows the characteristics of the study population by stroke status. The final sample consisted of 3,010 individuals, of whom 244 (7.53%) had experienced a stroke. The mean age of the included study population was 69.80 years, and the mean age of the stroke population was 73.02 years, with 1,513 (45.67%) males and 1,497 (54.33%) females. In the stroke population, 130 (43.54%) were male. Additionally, *p*-values calculated based on weighting showed statistically significant differences (*p* < 0.05) between age, education, hypertension, diabetes, physical activity, sedentary behavior, poverty income ratio, C-reactive protein, and stroke. There was no statistically significant association between gender, race, marital status, smoking status, drinking status, body mass index, and stroke (*p* > 0.05).

**Table 1:** Characterization of the study population by stroke status, weighted.

Variable	Total	Non-stroke	Stroke	*P*-value
*N(%)*	3010(100)	2,766(92.47)	244(7.53)	
Age (years)	69.80(69.41,70.20)	69.54(69.22,69.86)	73.02(71.46,74.59)	<0.0001
Gender, %				0.6772
Male	1,513(45.67)	1,383(45.84)	130(43.54)	
Female	1,497(54.33)	1,383(54.16)	114(54.46)	
Race, %				0.1347
Mexican American	388(4.04)	367(4.14)	21(2.86)	
Other Hispanic	253(2.54)	239(2.59)	14(1.93)	
Non-Hispanic White	1751(81.94)	1,603(82.17)	148(79.04)	
Non-Hispanic Black	531(7.95)	479(7.76)	52(10.29)	
Other Races	87(3.53)	78(3.34)	9(5.87)	
Education, %				0.0151
<High school	1,008(23.25)	914(22.67)	94(30.44)	
High school	727(26.09)	664(25.80)	63(29.65)	
>High school	1,275(50.66)	1,188(51.53)	87(39.91)	
Marital status, %				0.0612
Not living alone	1816(65.19)	1,677(65.76)	139(58.15)	
Living alone	1,194(34.81)	1,089(34.24)	105(41.85)	
Smoking status, %				0.4787
Smoker	1,563(51.29)	1,417(51.07)	146(53.92)	
Never smoker	1,447(48.71)	1,349(48.93)	98(46.08)	
Drinking status, %				0.1085
Yes	1930(66.35)	579(17.54)	85(31.50)	
No	1,080(33.65)	2,187(82.46)	159(68.50)	
Hypertension, %				<0.0001
Yes	1816(58.22)	1,614(56.34)	202(81.34)	
No	1,194(41.78)	1,152(43.66)	42(18.66)	
Diabetes, %				
Yes	664(18.59)	579(17.54)	85(31.50)	<0.0001
No	2,346(81.41)	2,187(82.46)	159(68.50)	
Poverty income ratio	3.01(2.90,3.12)	3.05(2.94,3.15)	2.56(2.33,2.79)	<0.0001
Body mass index	29.04(28.74,29.34)	29.04(28.73,29.36)	29.06(28.13,29.99)	0.9646
C-reactive protein (mg/dL)	0.44(0.40,0.48)	0.42(0.39,0.45)	0.69(0.46,0.92)	0.0234
Physical activity (MET-minutes/week)	1963.22(1727.17,2199.28)	2050.39(1810.15,2290.64)	893.03(607.21,1178.86)	<0.0001
Sedentary time (minutes)	352.00(339.58,364.41)	346.81(333.96,359.67)	415.66(381.21,450.11)	0.0006
Physical activity, %				<0.0001
<600 MET-minutes/week	1,651(51.46)	1,477(50.15)	174(67.44)	
≥600 MET-minutes/week	1,359(48.54)	1,289(49.85)	70(32.56)	
Sedentary behavior, %				0.0001
0 ~ <4 h/day	908(25.01)	861(25.78)	47(15.60)	
4 ~ <6 h/day	813(27.27)	758(27.62)	55(23.02)	
6 ~ 8 h/day	530(18.30)	479(18.34)	51(17.78)	
>8 h/day	759(29.42)	668(28.27)	91(43.60)	

For continuous variables: mean + SD, *P*-value was by survey-weighted linear regression; for categorical variables: N-observe (%), *P*-value was by survey-weighted Chi-square test.

### Weighted logistic regression results between sedentary behavior, physical activity and stroke

**Figure 2:**
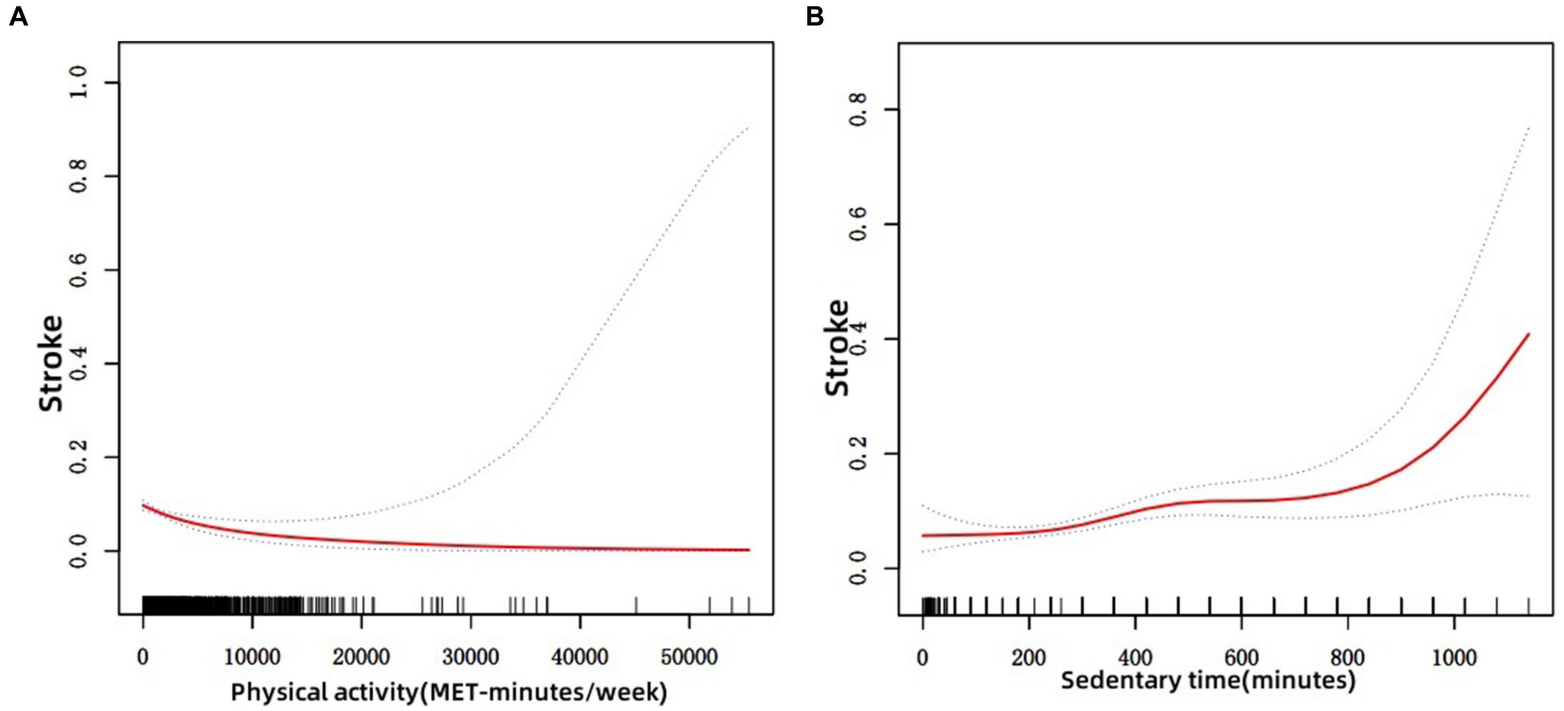
Results of a non-linear association between physical activity, sedentary time, and stroke in older adults. **(A)** Physical activity and stroke; **(B)** Sedentary time and stroke.

Table 2 shows the relationship between physical activity and stroke. <600 MET-minutes/week was used as the reference. In the crude model, the OR and 95% CI for stroke in older adults in the ≥600 MET-minutes/week group was 0.486 (0.360, 0.655); after adjusting for gender, age, and ethnicity, the OR and 95% CI for stroke in older adults was 0.548 (0.413, 0.727); when adjusted for all variables included, the results remained statistically significance [0.622 (0.443, 0.875), *p* = 0.009]. Table 3 shows the relationship between sedentary behavior and 60 years old, using 0 to <4 h/day as a reference. Sedentary behavior was found to be positively associated with the risk of 60-year-olds in the >8 h/day group in the crude model, with an OR and 95% CI of 2.549 (1.517, 4.284); the result was still significant after adjusting for all included variables [2.602 (1.557, 4.348), *p* = 0.003]. Figure 2 shows the results of smoothed curve fitting for the association of physical activity (A), sedentary behavior (B) and stroke. The smoothing curve shows a decrease in stroke risk as the amount of time spent exercising increases and then enters a plateau period. In contrast, the longer the sedentary time, the higher the risk of stroke, with a continuing upward trend.

**Table 2:** The relationship between physical activity and stroke in older adults, weighted.

	Crude model		Model 1		Model 2	
Physical activity	OR (95% CI)	*P*-value	OR (95% CI)	*P*-value	OR (95% CI)	*P*-value
Continuous	1.000(1.000,1.000)	0.002	1.000(1.000,1.000)	0.002	1.000(1.000,1.000)	0.006
<600 MET-minutes/week	Reference		Reference		Reference	
≥600 MET-minutes/week	0.486(0.360,0.655)	<0.001	0.548(0.413,0.727)	<0.001	0.622(0.443,0.875)	0.009

OR, odds ratio; CI, confidence intervals.

Crude model was unadjusted.

Model 1 is adjusted for gender, age, and race.

Model 2 was adjusted for gender, age, race, education, marital status, smoking status, drinking status, hypertension, diabetes, body mass index, and poverty income ratio.

**Table 3:** Relationship between sedentary behavior and stroke in older adults, weighted.

	Crude model		Model 1		Model 2	
Sedentary behavior	OR (95% CI)	*P*-value	OR (95% CI)	*P*-value	OR (95% CI)	*P*-value
Continuous	1.002(1.001,1.002)	<0.001	1.002(1.001,1.003)	<0.001	1.002(1.001,1.003)	<0.001
0 ~ <4 h/day	Reference		Reference		Reference	
4 ~ <6 h/day	1.378(0.863,2.198)	0.189	1.346(0.837,2.166)	0.233	1.275(0.798,2.035)	0.327
6 ~ 8 h/day	1.602(0.931,2.758)	0.100	1.422(0.807,2.506)	0.235	1.415(0.786,2.035)	0.267
>8 h/day	2.549(1.517,4.284)	0.001	2.615(1.527,4.480)	0.002	2.602(1.557,4.348)	0.003

OR, odds ratio; CI, confidence intervals.

Crude model was unadjusted.

Model 1 is adjusted for gender, age, and race.

Model 2 was adjusted for gender, age, race, education, marital status, smoking status, drinking status, hypertension, diabetes, body mass index, and poverty income ratio.

### Threshold effect analysis

Table 4 shows the results of the threshold effect analysis of the relationship between physical activity, sedentary behavior and stroke in older adults. The results showed that there was a threshold effect between physical activity, sedentary behavior and stroke, with thresholds of 480 MET minutes/week and 90 min for physical activity and sedentary behavior, respectively, and a significant threshold effect with stroke was found only for physical activity (Log-likelihood ratio < 0.001).

**Table 4:** Threshold effects between physical activity, sedentary behavior and stroke in older adults.

	Adjusted OR (95% CI), *P*-value
Physical activity	
Linear regression model	1.000(1.000,1.000),0.0037
Two-segment piecewise linear regression model	
Inflection point (K)	480
<K	0.999(0.998,0.999),0.0001
≥K	1.000(1.000,1.000),0.2999
Log-likelihood ratio	<0.001
Sedentary behavior	
Linear regression model	1.002(1.001,1.002),<0.0001
Two-segment piecewise linear regression model	
Inflection point (K)	90
<K	0.999(0.984,1.014),0.8921
≥K	1.002(1.001,1.002),<0.0001
Log-likelihood ratio	0.735

Adjusted for gender, age, race, education, marital status, smoking status, drinking status, hypertension, diabetes, body mass index, and poverty income ratio.

### Mediation analysis

**Figure 3:**
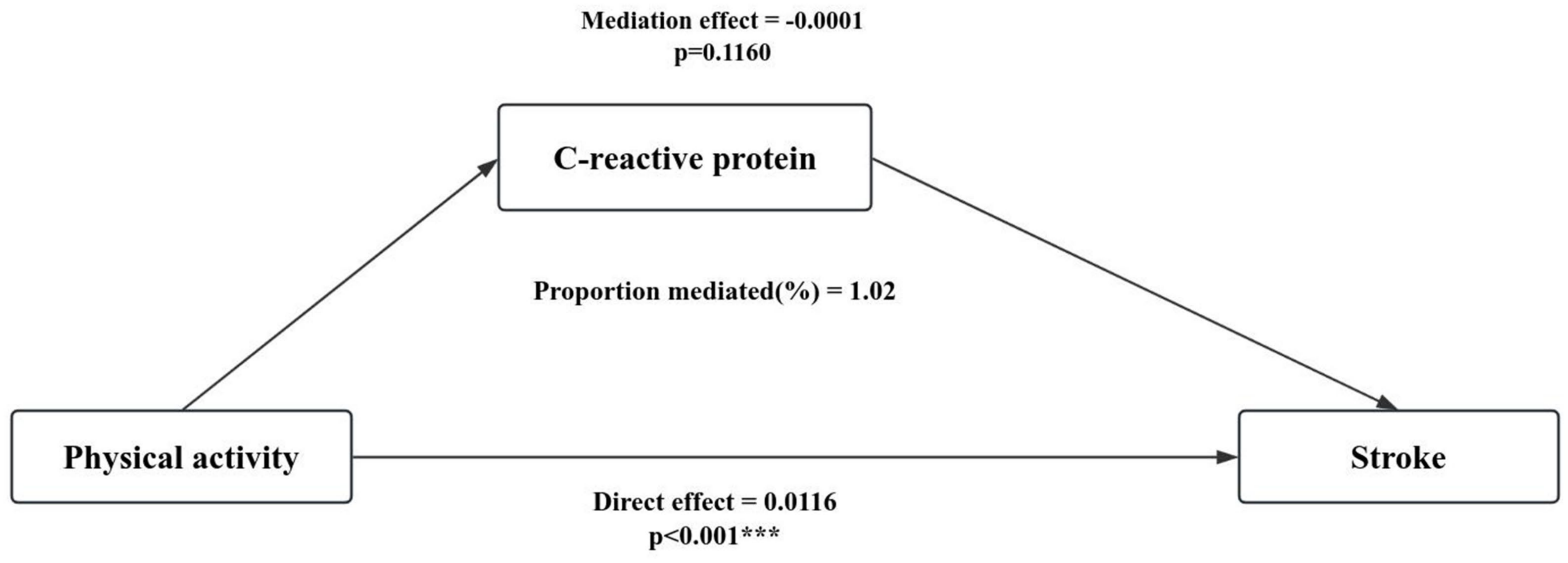
Mediation analysis of physical activity and stroke in older adults.

**Figure 4:**
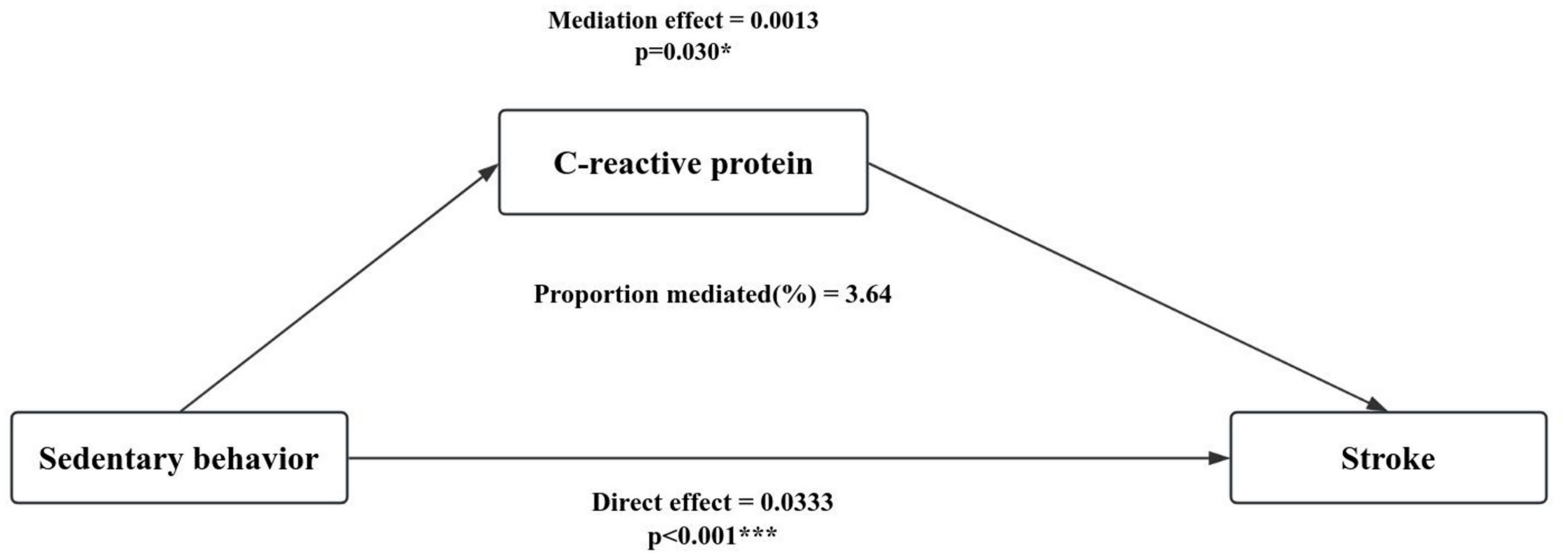
Mediation analysis of sedentary behavior and stroke in older adults.

Table 5 shows the weighted mean and 95% CI of C-reactive protein in different physical activity and sedentary behavior in older adults, with higher C-reactive protein levels in low physical activity levels and high sedentary behavior. The mediating role of C-reactive protein in the relationship between physical activity (Figure 3), sedentary behavior (Figure 4) and stroke in people aged 60 years and over was explored (sedentary behavior and physical activity were continuous variables). Adjusted for gender, age, race, education, marital status, smoking status, drinking status, hypertension, diabetes, body mass index, and poverty income ratio. A significant mediating effect (*p* < 0.05) was found only in sedentary behavior, with a mediation proportion of 3.64%.

**Table 5:** C-reactive protein levels in different physical activities, sedentary behavior.

	C-reactive protein (mg/dL)
Physical activity	
<600 MET-minutes/week	0.50(0.43,0.57)
≥600 MET-minutes/week	0.38(0.32,0.44)
Sedentary behavior	
0 ~ <4 h/day	0.35(0.31,0.39)
4 ~ <6 h/day	0.40(0.33,0.47)
6 ~ 8 h/day	0.51(0.41,0.61)
>8 h/day	0.52(0.44,0.59)

For continuous variables: survey-weighted mean (95% CI), *P*-value was by survey-weighted linear regression.

## Discussion

We found that the risk of stroke was lower among people aged 60 years and older who met the recommended exercise schedule of the US PA and that sedentary behavior was positively associated with the risk of stroke. C-reactive protein mediated the association between sedentary behavior and stroke in older adults with a mediator ratio of 3.64%.

Most studies have shown that physical activity reduces the risk of stroke. In a standardized case–control study of the global burden of stroke from various risk factors in 22 countries worldwide, the promotion of physical activity was found to significantly reduce the burden of stroke, [odds ratio (OR: 0.69, 0.53–0.90) and population-attributable risk (PAR: 28.5%, 14.5–48.5)]. In a study on stroke, it was noted that acute cerebral ischemia showed a distinct, slight pattern of intra-parenchymal hemorrhage differences in the onset time of weekdays versus weekends/holidays, which may be related to variations in physical activity and stressful times (21). The existing literature tends to suggest that sedentary behavior is associated with an increased risk of stroke. Sedentary behavior is linked to stroke risk. Among people under 60 years of age with low activity levels, recreational sedentary time over 8 h per day is associated with an increased risk of long-term stroke (4). A Sedentary lifestyle increases the risk of stroke (22), prolonged sedentary time is associated with more severe stroke symptoms (23).

Previous studies have found that inflammation appears to play a crucial role in the survival and recovery after ischemic stroke (24). Blood markers of inflammation are independently associated with vascular recurrence after stroke in a meta-analysis of individual participant data (10). In a study of the relationship between pro-inflammatory cytokines and the risk of recurrent stroke, it was demonstrated that inflammatory markers associated with the acute phase response (IL-6, TNF-*α*, C-reactive protein, and fibrinogen, but not IL-18), were associated with the risk of stroke recurrence (25). C-reactive protein has been shown to be a prognostic marker after lacunar stroke. In patients with recent lacunar stroke, high sensitivity C-reactive protein (hsCRP) levels predict the risk of recurrent stroke and other vascular events (26). Inflammation plays an important role in the pathophysiology of stroke, and it has been suggested that inflammatory risk markers within neutrophils, lymphocytes and C-reactive protein may have a strong independent predictive value for stroke outcome (27). The mediating effect of CRP explained only 3.64% of the relationship between sedentary behavior and stroke, which still suggests that sedentary behavior may affect stroke risk partly through inflammatory pathways. This finding suggests that sedentary behavior, even though a small inflammatory response, can still have a potentially negative impact on health. Therefore, it may serve as a basis for early identification and intervention in clinical settings.

Sedentary behavior is strongly associated with elevated levels of cytokines, which are also involved in many regulatory and inflammatory processes (28, 29). Previous studies have revealed the importance of cytokines in immune regulation, tissue repair and inflammatory diseases. In particular, the overexpression of some cytokines has been implicated in the pathogenesis of stroke (30), which makes exploring the relationship between sedentary behavior and cytokines an important direction in exploring the mechanisms of stroke genesis (31). This implies that sedentary behavior may influence the risk of stroke by affecting the degree of inflammatory response (32). The results of the mediation analyses suggest that C-reactive proteins play a significant regulatory role in the relationship between sedentary behavior and stroke. This finding triggered our interest in more in-depth studies on the role of inflammatory biomarkers in stroke pathogenesis. Further studies could explore the interactions between different inflammatory markers and how they play a role in sedentary behavior affecting stroke risk. This deeper dug information will help to understand the relationship between lifestyle and stroke more comprehensively and provide a more scientific basis for the development of prevention and intervention strategies.

Reducing sedentary behavior and increasing physical activity are effective ways to prevent stroke in older adults. A study of physical activity and stroke risk in middle-aged and older adults found negative associations between intensity, frequency, duration, and volume of physical activity and stroke risk in middle-aged and older adults (33). Physical activity is recognized as an effective intervention to improve psychosocial well-being after stroke (34). Exercise intervention programs improve frailty and cognitive traits, thereby optimizing functional capacity during the aging process, sedentary lifestyles are associated with declining muscle function and cardiorespiratory fitness, leading to impaired ability to perform daily activities and maintain independent functioning, and exercise is an alternative to medication (35).

Our study has several strengths. First, we used a nationally representative NHANES cohort, adjusted for confounders (including socio-demographic and lifestyle factors), and considered the complex sampling of NHANES in the analytical calculations. Secondly, the possible mediating role of inflammatory markers was considered when exploring the relationship between sedentary behavior, leisure physical activity and stroke, and C-reactive protein was found to significantly mediate the relationship between sedentary behavior and stroke in older adults. Finally, we also recognized that reducing sedentary time and increasing physical activity appeared to alter inflammatory status, and therefore, understanding the extent and specificity of the relationship between blood cell-based inflammatory biomarkers and stroke has further health implications.

There are several limitations to the study in this article. First, the data on physical activity and stroke came from a questionnaire, and the quantification of activity time by recall is obviously subjective, and more rigorous measurements, such as wearable physical activity monitor (PAM)-based physical activity measurements (36), should be adopted in subsequent studies; in addition, the physical activity equivalents calculated in this study were based on overall activity levels, and future studies could explore the effects of different types of physical activity levels on stroke (16); and finally, the source of this data is the United States, and caution should be exercised when extrapolating to the total population.

## Conclusion

In people aged 60 years and older, sedentary behavior was positively associated with stroke, whereas physical activity was negatively associated with stroke, and C-reactive protein mediated the relationship between sedentary behavior and stroke.

## Data Availability

The datasets presented in this study can be found in online repositories. The names of the repository/repositories and accession number(s) can be found: https://wwwn.cdc.gov/nchs/nhanes/Default.aspx.

## Footnotes

^1^ https://wwwn.cdc.gov/Nchs/Nhanes/2007–2008/PAQ_E.htm

^2^ http://www.empowerstats.com
